# Differential risk factors of fibrosis between lean and obese MAFLD

**DOI:** 10.1007/s10238-025-01749-1

**Published:** 2025-06-18

**Authors:** Alaa M. Mostafa, Yasser Fouad, Yasmine Gaber, Shereen Abdel Alem, Ziyan Pan, Mohammed Eslam

**Affiliations:** 1 Department of Gastroenterology, Hepatology and Endemic Medicine, Faculty of Medicine, Minia University, Main Road, Minia, 11432 Egypt; 2 Department of Endemic Medicine and Hepatology, Faculty of Medicine, Cairo University, Cairo, Egypt; 3 Storr Liver Centre, Westmead Institute for Medical Research, Westmead Hospital and University of Sydney, Sydney, NSW Australia

**Keywords:** MAFLD, Lean, Fatty, Fibrosis

## Abstract

Metabolic dysfunction-associated fatty liver disease (MAFLD) is often linked to overweight and obesity. However, a significant number of individuals with MAFLD are not obese, commonly referred to as lean MAFLD. This study aims to investigate the potential risk factors for fibrosis among lean individuals with MAFLD compared to those who are overweight or obese. The study included 7902 participants from the National Health and Nutrition Examination Survey (NHANES) data collected between 2017 and March 2020. MAFLD was defined in individuals with steatosis who were either overweight, diabetic, or lean and had at least two metabolic risk abnormalities. Demographic, anthropometric, and laboratory data, along with elastography results, were reported for each subject. Lean patients with MAFLD were significantly older (62.3 ± 13.8 years) compared to overweight or obese patients with MAFLD (51.7 ± 16.7 years; *p* < 0.001). Several factors were identified as predictors of significant fibrosis within the MAFLD population, including increasing age, BMI, ALT levels, alkaline phosphatase levels, lower platelet counts, lower HDL-cholesterol levels, and the presence of diabetes. In a multivariate logistic analysis of significant fibrosis (F > 2) in patients with obese MAFLD, female gender, diabetes, and hypertension were identified as risk factors. For lean individuals with MAFLD, older age, high AST levels, and lower platelet counts were found to be significant predictors of fibrosis. MAFLD among lean individuals is not a benign condition; those with metabolic dysfunction are at risk of developing fibrosis. The risk factors for fibrosis in these individuals may differ from those in their obese counterparts.

## Introduction

Metabolic dysfunction associated with fatty liver disease (MAFLD), formerly known as nonalcoholic fatty liver disease (NAFLD), is an increasingly prevalent chronic liver disease globally and has emerged as a substantial public health issue due to its rising rates, which have doubled over the last 20 years [1, 2]. MAFLD encompasses a broad spectrum of liver damage, ranging from simple steatosis to steatohepatitis, advanced fibrosis, cirrhosis, and liver cancer [3, 4]. MAFLD is diagnosed when two or more metabolic risk abnormalities are present, with overweight/obesity or type 2 diabetes (T2DM) suggested as straightforward diagnostic criteria [5].

Overweight and obesity are common clinical phenotypes associated with MAFLD. Individuals with MAFLD who do not meet the criteria for overweight or obesity are classified as having lean MAFLD, based on ethnic-specific BMI classifications [5, 6]. Although it is often believed that individuals with lean MAFLD experience a more benign clinical course compared to those who are overweight or obese—due to their typically better metabolic and histologic profiles mediated by differential metabolic adaptation—paradoxically, they tend to have worse long-term outcomes and higher mortality rates [7–9]. This may be related to differences in telomere length [10].

There is still a significant lack of understanding of the lean MAFLD profile. There is significant scientific interest in understanding how it differs from non-lean MAFLD in terms of features, risk factors, outcomes, and management. Therefore, the aim of our study is to investigate the potential risk factors associated with MAFLD in lean individuals compared to those who are overweight or obese.

## Methods

The cohort of this study was extracted from the National Health and Nutrition Examination Survey (NHANES) 2017-March 2020 data documentation, codebook, and frequencies. This survey is a part of the Centers for Disease Control and Prevention archiving program, which aims to assess people's nutritional and overall health in the United States [11].

### Study population

Patients who fulfilled the following criteria were considered suitable for inclusion in this study: age ≥ 18 years old), having complete transient elastography (TE) examination. The following criteria were used to exclude patients: age < 18 years old, absent CAP results, alcohol intake (> 2 drinks per day for men and > 1 per day for women every or nearly every day), positive hepatitis C confirmed by HCV RNA, and positive hepatitis B surface antigen HBs Ag.

### Patients characterization

Demographic data: age, gender, and race were obtained for all studied groups. We defined the MAFLD population according to CAP ≥ 264 with metabolic criteria: overweight or obesity (defined by BMI ≥ 25 mg/m^2^/≥ 23 mg/m^2^ in Asian), T2DM, or lean/normal weight with at least two metabolic risk abnormalities; (Waist circumference ≥ 102/88 cm in Caucasian men and women or 90/80 cm in Asian men and women), (blood pressure ≥ 130/85 or told by the doctor to have hypertension), (Plasma Triglyceride level is ≥ 1.7 mmol/dL), (Plasma HDL-Cholesterol level < 1.0/1.3 mmol/dL for men and women). HOMA-IR ≥ 2.5, and Hs-CRP ≥ 2 mg/L. Prediabetes and diabetes were defined according to the questionnaire (DIQ010 doctors had told you to have diabetes).

### Vibration-controlled transient elastography (VCTA)

Liver Ultrasound Transient Elastography Procedures Manuals of this component. The elastography measurements were obtained in the NHANES Mobile Examination Center (MEC), using the FibroScan® model 502 V2 Touch equipped with a medium (M) or extra-large (XL) wand (probe). A median CAP cut-off of ≥ 264 dB/m and ≥ 8 kPa is used to define steatosis S1 (CAP) and significant fibrosis F2 (LSM) [4, 12].

### Anthropometric and Laboratory data

**Fig. 1:**
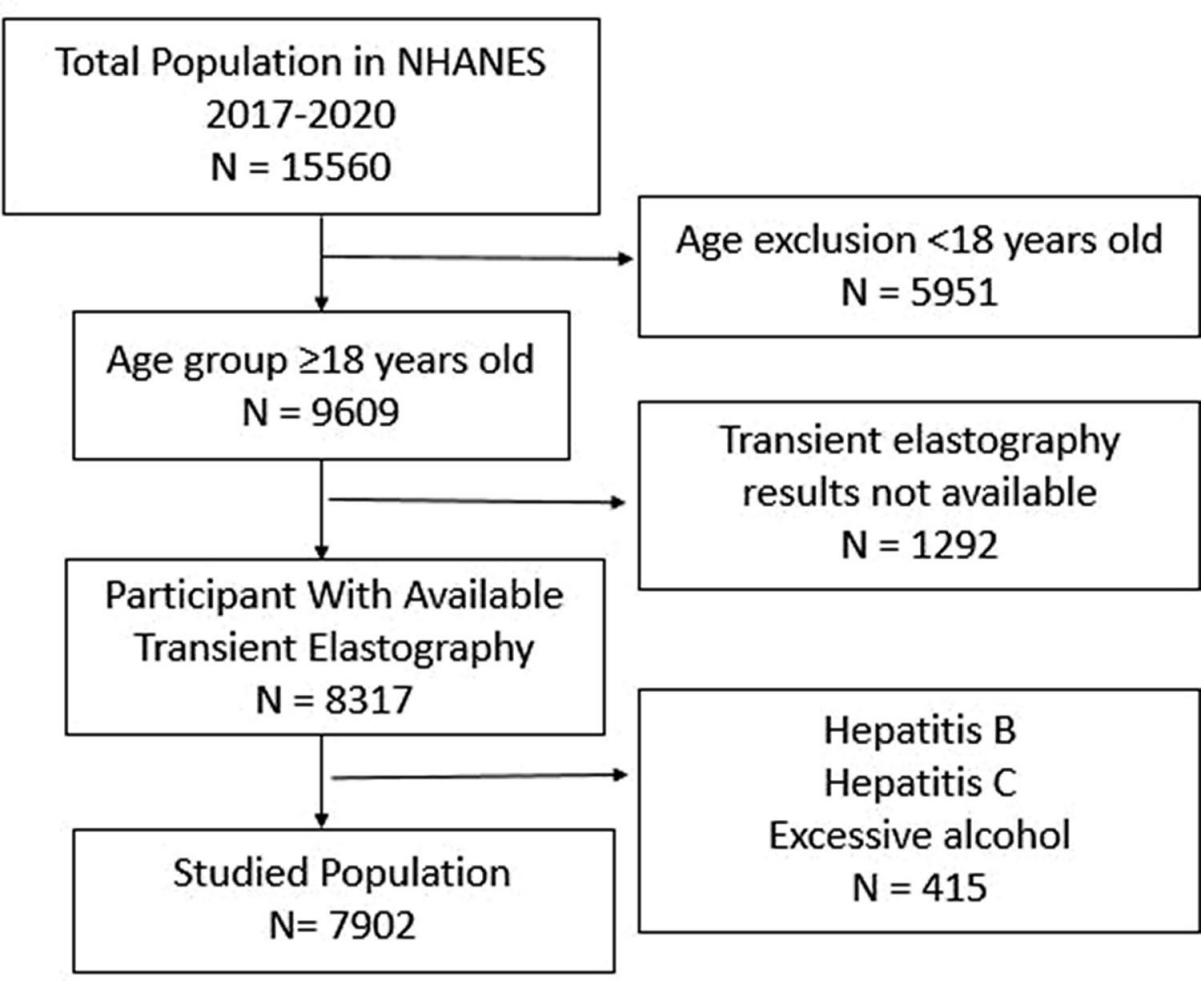
Flow chart of study population selection

The following information was all obtained from examination and laboratory measurements. Body mass index BMI, waist circumference WC, blood pressure, dyslipidemia, and current alcohol intake. Laboratory information includes fasting blood sugar FBS, HbA1C, hemostasis model assessment of insulin resistance score HOMA-IR, plasma high sensitivity C-reactive protein level Hs-CRP, Cholesterol, Triglyceride, low-density lipoprotein LDL, high-density lipoprotein HDL, hemoglobin level Hb, total leucocyte count TLC, Platelet count, Alanine transferase ALT, Aspartate transferase AST, Serum albumin, total protein, S uric acid, alkaline phosphatase, serum creatinine, and insulin level were obtained. Fibrosis-4 index (FIB-4) and NAFLD fibrosis score NFS were calculated (Fig. 1).

### Statistical analysis

Data cleaning was done first to ensure our sample size satisfied the diagnostic criteria for MAFLD (TE and metabolic criteria). We categorized the MAFLD population according to BMI and/or the presence of significant fibrosis (F2) and compared the distribution of demographic characteristics, metabolic criteria, and laboratory findings in each group. The continuous variables, described by mean ± SD, were assessed by Student’s t-test. Categorical variables accounted by number (%) and evaluated using the chi-square test. We performed multivariate logistic regression analysis to estimate risk factors of significant fibrosis (F ≥ 2) in MAFLD, obese, and lean MAFLD. The results were addressed by odds ratio (OR) and 95% CI. Statistical significance was indicated as (*p* < 0.05).

## Results

After applying exclusion criteria, 7902 subjects remained in the study, among which 3810 (48%) fit the MAFLD criteria. Patients with MAFLD were older and leaning towards the male gender. Descriptive statistics are reported in Table 1.

**Table 1:** Descriptive data of the studied population

		All N 7902	Not MAFLD N 4092	MAFLD N 3810
Age (18–80)		49.01 ± 18.4	46.1 ± 19.3	52.1 ± 16.8
Sex	Male	3848(48.7)	1833 (44.8)	2015 (52.9)
	Female	4053 (51.3)	2258 (55.2)	1795 (47.1)
Smoking	1278(41.9)	717 (17.5)	561 (14.7)	
Race	Mexican American	977 (12.4)	373 (9.1)	604 (15.9)
	Other Hispanic	840 (10.6)	425 (10.4)	415 (10.9)
	Non-Hispanic White	2684 (34)	1341(32.8)	1343 (35.2)
	Non-Hispanic Black	2046(25.9)	1197 (29.3)	849 (22.3)
	Asian	967 (12.2)	548(13.4)	419 (10.9)
	Other race	388 (4.9)	208 (4.2)	180 (4.7)
BMI (Kg/mm^2^)		28.9 ± 7.5	26.3 ± 5.7	33.7 ± 7.4
WC (cm)		100.2 ± 17.4	90.9 ± 13.8	110.2 ± 15.3
Diabetic		1138 (23.8)	312 (7.6)	826 (21.7)
Prediabetes		231 (2.9)	79 (1.9)	152 (4)
FBS (mmHg)		6.3 ± 2.1	5.8 ± 1.5	6.8 ± 2.5
HBAIC		5.8 ± 1.1	5.6 ± 0.8	6.1 ± 1.3
HOMA-IR		4.5 ± 8.2	2.7 ± 6.3	6.3 ± 9.5
Hypertension		4204 (56.7)	1748 (42.7)	2456 (64.5)
Systolic blood pressure (mmHg)		124.1 ± 19.6	121.7 ± 19.8	126.5 ± 18.9
Diastolic blood pressure (mmHg)		74.6 ± 11.7	72 ± 11.2	76.4 ± 11.7
HS-CRP (mg/L)		4 ± 8.5	3.04 ± 7.5	5.07 ± 9.3
Dyslipidemia		2485 (33.7)	823 (20.1)	1662 (43.6)
Triglyceride (mmol/L)		1.6 ± 1.2	1.2 ± 0.8	1.9 ± 1.4
Cholesterol (mmol/L)		4.7 ± 1.04	4.6 ± 1.02	4.8 ± 1.1
LDL (mmol/L)		2.8 ± 0.9	2.8 ± 0.9	2.9 ± 0.9
HDL (mmol/L)		1.4 ± 0.4	1.5 ± 0.4	1.2 ± 0.4
CAP ≥ 264		3914 (49.5)	104	3810
CAP dB/m		263.7 ± 63.2	214.4 ± 36.2	316.6 ± 38.2
LSM F ≥ 2		855 (10.8)	180 (4.4)	675 (17.7)
LSM (kPa)		5.9 ± 5.1	5.1 ± 3.9	6.9 ± 5.9
Hemoglobin (g/dL)		13.9 ± 1.6	13.8 ± 1.5	14.2 ± 1.6
Total Leucocyte count (cell/mm3)		7.2 ± 5.1	6.9 ± 6.7	7.6 ± 2.1
Platelet (109/L)		247.5 ± 65.2	244.4 ± 63.9	250.7 ± 66.4
ALT (U/L)		21.6 ± 16.2	17.9 ± 13.1	25.5 ± 18.1
AST (U/L)		21.2 ± 11.7	20.2 ± 10.4	22.3 ± 12.7
S albumin (g/dL)		4.1 ± 0.3	4.1 ± 0.3	4 ± 0.3
Total protein (g/dL)		7.2 ± 0.4	7.2 ± 0.5	7.2 ± 0.4
Total Bilirubin (g/dL)		0.4 ± 0.3	0.5 ± 0.3	0.4 ± 0.3
S. Creatinine (mg/dL)		0.9 ± 0.5	0.9 ± 0.4	0.9 ± 0.5
Alkaline phosphatase (IU/L)		78.04 ± 25.9	74.6 ± 25.9	81.6 ± 225.5
Insulin level (µU/mL)		14.8 ± 22	9.9 ± 15.9	19.9 ± 25.9
Uric acid (mg/dL)		5.4 ± 1.5	5.03 ± 1.3	5.8 ± 1.5
FIB-4		1.05 ± 0.8	1.03 ± 0.7	1.07 ± 0.9
NFS		− 1.6 ± 1.6	− 2.04 ± 1.5	− 1.2 ± 1.6

BMI: body mass index; WC: waist circumference; FBS: fasting blood sugar; HbA1C: glycated hemoglobin; HOMA-IR: homeostatic model assessment for insulin resistance; HS-CRP: high sensitivity C-reactive protein test; LDL: low density lipoprotein, HDL: high-density lipoprotein; CAP: controlled attenuation parameter; LSM: liver stiffness measurement; ALT: alanine transferase; AST: aspartate transferase; FIB-4: fibrosis-4 index; NFS: NAFLD fibrosis score

Regarding the lean MAFLD prevalence, 3.6% of patients with MAFLD were lean. Lean MAFLD patients were significantly older (62.3 ± 13.8 vs. 51.7 ± 16.7 years; *p* < 0.001) than overweight or obese MAFLD. There was no significant gender or racial distribution between the studied groups except for a higher prevalence of obese non-Hispanic Black (22.6 vs. 15.2% lean MAFLD) and lean Asian (21 vs. 10.6% obese MAFLD; *p* 0.002). Overweight/obese MAFLD patients have significantly lower blood pressure (63.9 vs. 79%; *p* < 0.001), higher HOMA-IR levels (6.4 ± 9.3 vs. 4.7 ± 13.9; *p* < 0.001), and higher Hs-CRP (6.4 ± 9.3 vs 3.7 ± 7.3 mg/L; *p* < 0.001) than lean MAFLD. A significant reduction in cholesterol and HDL-cholesterol levels was notable in patients with overweight/obese MAFLD (4.8 ± 1.05 mmol/L; *p*-value 0.008 and 1.2 ± 0.3 mmol/L; *p* < 0.001) compared with those with lean MAFLD (Table 2).

**Table 2:** Comparison of lean versus overweight/obese MAFLD

		Lean MAFLD N 138	Overweight/obese MAFLD N 3644	*p*-Value
Age		62.3 ± 13.8	51.7 ± 16.7	< 0.001
Male		73 (52.9)	1925 (52.8)	0.9
Race	Mexican American	16 (11.6)	582 (15.9)	0.002
	Other Hispanic	12 (8.7)	383 (10.5)	
	Non-Hispanic White	52 (37.7)	1282 (35.2)	
	Non-Hispanic Black	21(15.2)	823 (22.6)	
	Non-Hispanic Asian	29 (21)	387 (10.6)	
	Other	8 (5.8)	171 (4.7)	
Smoking		22 (15.9	537 (14.7)	0.8
Diabetes		33 (23.9)	782 (21.5)	0.5
Prediabetic		9 (6.5)	142 (3.9)	0.1
FBS (mmHg)		6.9 ± 2.9	6.8 ± 2.4	0.9
HbA1C (mmoL)		6.2 ± 1.5	6.1 ± 1.3	0.5
HOMAIR		4.7 ± 13.9	6.4 ± 9.3	< 0.001
Hs-CRP (mg/L)		3.7 ± 7.3	5.1 ± 9.4	< 0.001
Hypertension		109 (78.9)	2327 (63.9)	< 0.001
Dyslipidemia		55 (39.8)	1596 (43.8)	0.3
Triglyceride (mmol/L)		1.9 ± 1.11	1.9 ± 1.4	0.9
Cholesterol (mmol/L)		5.1 ± 1.3	4.8 ± 1.05	0.008
LDL-cholesterol (mmol/L)		3.07 ± 1.07	2.9 ± 0.9	0.2
HDL-cholesterol (mmol/L)		1.4 ± 0.6	1.2 ± 0.3	< 0.001
CAP dB/m		299.3 ± 27.6	317.3 ± 38.5	< 0.001
LSM		9 (6.5)	660 (18.1)	< 0.001
LSM (kPa)		5.2 ± 3.6	6.9 ± 6.03	< 0.001
Platelets (109/L)		230.7 ± 66.9	251.6 ± 66.4	< 0.001
ALT (IU/L)		20.1 ± 12.3	25.8 ± 18.3	< 0.001
AST (IU/L)		22.3 ± 11.9	22.3 ± 12.6	0.7
S Albumin (g/dL)		4.2 ± 0.3	4.03 ± 0.3	< 0.001
T protein (g/dL)		7.24 ± 0.45	7.16 ± 0.43	0.05
S creatinine (mg/dL)		0.9 ± 0.3	0.9 ± 6.05	0.9
Alkaline Phosphatase (IU/L)		84.7 ± 27.01	81.4 ± 25.4	0.1
Insulin (µU/mL)		11.9 ± 14.6	20.3 ± 26.3	< 0.001
Uric acid (mg/dL)		5.5 ± 1.5	5.8 ± 1.5	0.04
FIB-4		1.7 ± 3.2	1.04 ± 0.7	< 0.001
NFS		− 1.4 ± 1.4	− 1.2 ± 1.6	0.13

FBS: fasting blood sugar; HbA1C: glycated hemoglobin; HOMA-IR: homeostatic model assessment for insulin resistance; HS-CRP: high sensitivity C-reactive protein test; LDL: low density lipoprotein, HDL: high-density lipoprotein; CAP: controlled attenuation parameter; LSM: liver stiffness measurement; ALT: alanine transferase; AST: aspartate transferase; FIB-4: fibrosis-4 index; NFS: NAFLD fibrosis score

Considering the impact of fibrosis, we classified all patients with MAFLD into two groups according to the presence of significant fibrosis (LSM ≥ 8), which was found in 17.7% of MAFLD patients (Table 3). MAFLD with significant fibrosis tend to be older (54.3 ± 15.6 vs 51.7 ± 16.9 years; *p* < 0.001) with a higher proportion of males 57.04%; *p* 0.02. Patients with substantial fibrosis have shown a higher prevalence of diabetes, hypertension, and dyslipidemia. A notable statistical association was found between significant fibrosis and other factors, including higher BMI, waist circumference, FBS, HbA1C, HOMA-IR, Hs-CRP, Triglyceride, ALT, AST, alkaline phosphatase, Uric Acid, NFS, and degree of steatosis. A lower platelet, HDL-cholesterol, and S albumin were statistically associated with significant fibrosis.

**Table 3:** Comparing patients with fibrosis versus those without fibrosis in patients with MAFLD

		LSM < 8 N 3135	LSM ≥ 8 N 675	*p*-Value
	Age	51.7 ± 16.9	54.3 ± 15.6	< 0.001
	Male	1630 (51.9)	385 (57.04)	0.02
Race	Mexican American	504 (16.1)	100(14.8)	0.6
	Other Hispanic	348(11.1)	67(9.9)	
	Non-Hispanic White	1084(34.6)	259(38.4)	
	Non-Hispanic Black	690(22)	159(23.6)	
	Non-Hispanic Asian	364(11.6)	55(8.1)	
	Other	145(4.6)	35(5.2)	
	Smoking	467(14.9)	94(13.9)	0.07
	BMI (Kg/mm2)	32.5 ± 6.3	38.8 ± 9.4	< 0.001
	WC (cm)	107.7 ± 13.6	121.9 ± 17.1	< 0.001
	Diabetes	577 (18.4)	249 (36.9)	< 0.001
	Prediabetic	123 (3.9)	29(4.3)	0.7
	FBS (mmHg)	6.6 ± 2.2	7.9 ± 3.3	< 0.001
	HbA1C	6.02 ± 1.2	6.6 ± 1.6	< 0.001
	HOMAIR	5.6 ± 7.7	10.1 ± 15.2	< 0.001
	Hs-CRP (mg/L)	4.7 ± 9.1	6.6 ± 10.1	< 0.001
	Hypertension	1940 (61.9)	516 (76.4)	< 0.001
	Dyslipidemia	1332 (42.5)	330 (49)	0.001
	Triglyceride (mmol/L)	1.85 ± 1.3	2.02 ± 1.6	0.002
	Cholesterol (mmol/L)	4.9 ± 1.05	4.7 ± 1.08	< 0.001
	LDL-cholesterol (mmol/L)	2.9 ± 0.9	2.8 ± 0.9	< 0.001
	HDL-cholesterol (mmol/L)	1.26 ± 0.35	1.19 ± 0.35	< 0.001
	Platelets (109/L)	252.3 ± 64.7	243.3 ± 73.6	< 0.001
	ALT (IU/L)	24.4 ± 16	30.8 ± 25.1	< 0.001
	AST (IU/L)	21.3 ± 9.5	26.8 ± 21.6	< 0.001
	S Albumin (g/dL)	4.05 ± 0.3	3.9 ± 0.3	< 0.001
	Total protein (g/dL)	7.2 ± 0.4	7.2 ± 0.5	0.7
	Total bilirubin (g/dL)	0.4 ± 0.2	0.5 ± 0.3	0.008
	S creatinine (mg/dL)	0.9 ± 0.4	0.9 ± 0.7	0.09
	Alkaline Phosphatase (IU/L)	80.6 ± 24.1	86.5 ± 30.9	< 0.001
	Insulin (µU/mL)	18.3 ± 19.9	28.3 ± 44.2	< 0.001
	Uric acid (mg/dL)	5.7 ± 1.4	6.1 ± 1.6	< 0.001
	CAP dB/m	312.01 ± 36.1	338.1 ± 40.6	< 0.001
	FIB-4	1.02 ± 0.9	1.3 ± 1.04	< 0.001
	NFS	− 1.4 ± 1.5	− 0.27 ± 1.6	< 0.001

BMI: body mass index; WC: waist circumference; FBS: fasting blood sugar; HbA1C: glycated hemoglobin; HOMA-IR: homeostatic model assessment for insulin resistance; HS-CRP: high sensitivity C-reactive protein test; LDL: low density lipoprotein, HDL: high-density lipoprotein; CAP: controlled attenuation parameter; LSM: liver stiffness measurement; ALT: alanine transferase; AST: aspartate transferase; FIB-4: fibrosis-4 index; NFS: NAFLD fibrosis score

When we stratified lean and obese MAFLD by liver stiffness measurement (LSM) (Table 4), Overweight/obese and lean MAFLD tended to be older (54.2 ± 16.5 and 66.1 ± 11.8 years respectively), with statistical significance in obese MAFLD (*p* < 0.001). A potential gender-related variation in the percentage of males in the obese MAFLD with LSM ≥ 8 was substantially higher (57.6 vs. 51.8%; *p* 0.007), while no significant conclusions can be drawn due to the small sample size in lean MAFLD of LSM ≥ 8. Both groups of obese and lean MAFLD show more metabolic risk factors with significant association in the obese MAFLD group with higher WC, DM, FBS, HbA1C, HOMA-IR, triglyceride, CAP, ALT, AST, Alkaline phosphatase, insulin level, S Uric acid. In lean MAFLD, significant fibrosis is statistically associated with Hs-CRP, platelet, AST, and Alkaline phosphatase.

**Table 4:** Risk factors for fibrosis according to LSM in lean and obese MAFLD

	Lean MAFLD N 138		*p*-Value	Overweight/obese MAFLD N 3644		*p*-Value
	LSM < 8	LSM ≥ 8		LSM < 8	LSM ≥ 8	
	N 129	N 9		N 2984	N 660	
Age	61.9 ± 13.9	66.1 ± 11.8	0.52	51.2 ± 16.9	54.2 ± 16.5	< 0.001
Male	70 (54.3)	3 (33.3)	–	1545 (51.8)	380 (57.6)	0.007
Smoking	19 (14.7)	3 (33.3)	–	447 (14.9)	90 (13.6)	0.05
WC	88.7 ± 6.8	91.8 ± 7.9	0.2	108.5 ± 13.2	122.4 ± 16.9	< 0.001
Diabetes	28 (21.7)	5 (55.6)	–	541 (18.1)	241 (36.5)	< 0.001
FBS	6.8 ± 2.7	8.5 ± 4.6	0.4	6.6 ± 2.1	7.9 ± 3.3	< 0.001
HbA1C	6.2 ± 1.5	6.8 ± 1.5	0.2	6.01 ± 1.2	6.6 ± 1.6	< 0.001
HOMAIR	4.7 ± 14.3	5.4 ± 5.7	0.8	5.6 ± 7.1	10.2 ± 15.3	< 0.001
HS-CRP	3.5 ± 7.1	6.4 ± 9.8	0.03	4.8 ± 9.2	6.6 ± 10.2	< 0.001
Hypertension	101 (78.3)	8 (88.9)	0.6	1822 (61.1)	505 (76.5)	< 0.001
Dyslipidemia	50 (38.8)	5 (55.6)	–	1274 (42.7)	322 (48.8)	0.002
Triglyceride	1.8 ± 1.09	2.1 ± 1.4	0.8	1.9 ± 1.4	2.02 ± 1.6	0.004
Cholesterol	5.2 ± 1.2	4.7 ± 1.8	0.3	4.9 ± 1.04	4.7 ± 1.06	< 0.001
LDL	3.1 ± 1.07	2.5 ± 1.05	0.2	2.9 ± 0.9	2.8 ± 0.9	< 0.001
HDL	1.4 ± 0.6	1.5 ± 0.7	0.9	1.3 ± 0.3	1.2 ± 0.3	< 0.001
CAP	298.6 ± 26.9	309.4 ± 36.5	0.4	312.6 ± 36.4	338.5 ± 40.6	< 0.001
Hemoglobin	14.1 ± 1.4	13.5 ± 2.2	0.3	14.2 ± 1.5	14.2 ± 1.6	0.7
TLC	7.04 ± 2.2	7.6 ± 2.6	0.4	7.5 ± 2.1	7.9 ± 2.3	< 0.001
Platelets	227.7 ± 62.9	274 ± 104.4	0.045	253.4 ± 64.6	242.9 ± 73.4	< 0.001
ALT	20 ± 11.4	26.6 ± 20.5	0.3	24.6 ± 16.2	30.9 ± 25.2	< 0.001
AST	21.7 ± 11.2	31.1 ± 17.9	0.048	21.4 ± 9.5	26.6 ± 21.3	< 0.001
S Albumin	4.2 ± 0.3	4.1 ± 0.5	0.8	4.05 ± 0.3	3.9 ± 0.3	< 0.001
T protein	7.2 ± 0.5	7.2 ± 0.6	0.8	7.2 ± 0.4	7.2 ± 0.5	0.7
T bilirubin	0.5 ± 0.3	0.6 ± 0.4	0.6	0.4 ± 0.3	0.5 ± 0.3	0.003
S creatinine	0.8 ± 0.2	0.8 ± 0.3	0.3	0.8 ± 0.4	0.9 ± 0.7	0.047
Alkaline Phosphatase	83.5 ± 26.7	100.7 ± 27.01	0.02	80.4 ± 23.9	85.9 ± 30.8	< 0.001
Insulin	11.9 ± 14.9	12.1 ± 6.3	0.6	18.6 ± 19.9	28.7 ± 44.9	< 0.001
Uric acid	5.5 ± 1.5	6 ± 1.5	0.3	5.6 ± 1.4	6.1 ± 1.7	< 0.001
FIB-4	1.7 ± 3.3	1.7 ± 1.07	0.8	0.99 ± 0.58	1.3 ± 1.02	< 0.001
NFS	− 1.4 ± 1.4	− 1.3 ± 1.7	0.9	− 1.4 ± 1.5	− 0.2 ± 1.6	< 0.001

WC: waist circumference; FBS: fasting blood sugar; HbA1C: glycated hemoglobin; HOMA-IR: homeostatic model assessment for insulin resistance; HS-CRP: high sensitivity C-reactive protein test; LDL: low density lipoprotein, HDL: high-density lipoprotein; TLC: total leucocyte count; ALT: alanine transferase; AST: aspartate transferase; CAP: controlled attenuation parameter; LSM: liver stiffness measurement; FIB-4: fibrosis-4 index; NFS: NAFLD fibrosis score

We used a logistic regression analysis to highlight the Key liver-related and metabolic parameters that predict the presence of significant fibrosis in MAFLD patients. Increasing age, BMI, ALT, and Alkaline phosphatase were predictors of significant fibrosis in the MAFLD population. Additionally, lower levels of platelet and HDL-cholesterol were indicators for a higher risk of fibrosis in the MAFLD population. Diabetes was a strong predictor of fibrosis in MAFLD patients. (Table 5).

**Table 5:** Multivariate Logistic regression analysis predicting significant fibrosis in MAFLD population (F ≥ 2)

	OR	95% CI		*p*-value
Age	1.02	1.01	1.03	< 0.001
Gender (Male)	0.8	0.63	1.02	0.07
BMI	1.13	1.12	1.15	< 0.001
Hypertension	1.28	0.99	1.65	0.05
Diabetes	2.12	1.68	2.65	< 0.001
Platelet	0.99	0.99	0.99	0.005
ALT	1.02	1.02	1.03	< 0.001
Alkaline Phosphatase	1.01	1.002	1.009	0.001
HDL	0.69	0.49	0.97	0.03
Uric acid	1.05	0.98	1.13	0.14
HS-CRP	1.001	0.99	1.01	0.8

BMI: body mass index; ALT: alanine transferase; HDL: high-density lipoprotein; HS-CRP: high sensitivity C-reactive protein test

Using a multivariate logistic analysis predicting significant fibrosis (F > 2) in patients with obese MAFLD, gender, the presence of diabetes and hypertension, HDL-cholesterol, platelets, ALT, Uric acid, and Alkaline phosphatase were risk factors of fibrosis. (Table 6).

**Table 6:** Multivariate logistic regression analysis Predicting Significant Fibrosis in Obese MAFLD Using F ≥ 2

	Obese MAFLD			
	OR	95% CI		*p*-value
Age	1.00	0.99	1.01	0.2
Gender (Male)	0.76	0.6	0.9	0.01
Diabetes	2.3	1.86	2.86	< 0.001
Hypertension	1.6	1.2	1.97	< 0.001
Hs-CRP	1.01	1.003	1.02	0.008
HDL-cholesterol	0.56	0.41	0.78	< 0.001
Platelet	0.99	0.99	0.99	0.009
ALT	1.02	1.01	1.02	< 0.001
S Uric acid	1.18	1.11	1.26	< 0.001
Alkaline Phosphatase	1.005	1.002	1.009	0.001

HS-CRP: high-sensitivity C-reactive protein test; HDL-cholesterol: high-density lipoprotein; ALT: alanine transferase

The logistic regression for key metabolic risk factors predicting significant fibrosis in lean MAFLD did not show strong independent predictors for fibrosis in lean MAFLD. (Table 7). When using Fib-4 ≥ 1.3 to identify significant predictors of fibrosis in lean MAFLD, older Age, high AST, and lower platelet count were predictors of significant fibrosis in lean MAFLD. (Table 8).

**Table 7:** Multivariate logistic regression analysis Predicting Significant Fibrosis in Lean MAFLD Using F ≥ 2

	OR	95% CI		*p*-Value
AST	1.03	0.99	1.07	0.1
Platelet	1.01	0.99	1.02	0.08
Alkaline phosphatase	1.02	0.99	1.02	0.2
HS-CRP	1.01	0.94	1.08	0.8

AST: aspartate transferase; Hs-CRP: high sensitivity C-reactive protein

**Table 8:** Logistic Regression Analysis Predicting Significant Fibrosis in Lean MAFLD Using Fib-4

	OR	95% CI		P-value	OR	95% CI		*p*-Value
Age	1.09	1.05	1.13	< 0.001	1.28	1.15	1.42	< 0.001
AST	1.05	1.001	1.1	0.04	1.21	1.10	1.32	< 0.001
Platelet	0.97	0.96	0.98	< 0.001	0.94	0.91	0.96	< 0.001

AST: aspartate transferase

## Discussion

MAFLD is extensively researched in obese and overweight patients; however, there is a unique challenge in lean individuals due to a scarcity of data regarding lean-MAFLD (L-MAFLD) risk factors, pathogenesis, fibrosis progression, diagnosis, and management [13]. This study assessed the different risk factors for fibrosis in lean and obese MAFLD, together with predictors of significant fibrosis in MAFLD patients.

There is a wide prevalence of Lean MAFLD across populations. In this study, using a nationwide representative sample of the United States (US) population, the overall prevalence of L-MAFLD was 3.6%, more seen in white non-Hispanics, this was lower than that reported in different studies done in the US, with the prevalence rate of L-NAFLD ranged from 7 to 12% [14, 15], however, another study reported a broader range of prevalence of L-NAFLD (5–26%) in the adult population worldwide [16], this variation could be due to difference in the sensitivity and specificity of diagnostic methods of MAFLD, ethnicity, and the old exclusion criteria of NAFLD diagnosis, in our study most of the population are non-Hispanic, however, L-MAFLD was reported higher in Asian [17].

Lean individuals with MAFLD have a different clinical profile than overweight/obese individuals. The current study showed that lean MAFLD individuals were significantly older than obese-MAFLD, and interestingly they had some components of metabolic risk factors, including more hypertension, and hypercholesterolemia than obese-MAFLD, however, they had lower IR, HS-CRP, and higher HDL-cholesterol levels. This finding agreed with studies that reported L-NAFLD individuals tended to be older [18, 19], and despite several studies showing that L-NAFLD patients were less likely to have components of metabolic syndrome than obese and overweight NAFLD [15, 18, 19] other studies agreed with our results that L-MAFLD is considered as metabolically unhealthy status and closely linked with various metabolic diseases including metabolic syndrome [20, 21]. However, a recent meta-analysis showed that obese patients had more diabetes mellitus and other metabolic diseases compared to those with L-MAFLD [22]. Consistent with the hypercholesterolemia that was reported in our L-MAFLD patients, a recent large retrospective study investigating the metabolic syndrome in the L-NAFLD population showed that total cholesterol was significantly higher in the L-NAFLD than overweight/obese NAFLD [23], which was similar to the results of another study [24]. Although individuals with L-NAFLD tending to have less severe diseases, nominal insulin resistance, and dyslipidemia, these conditions are still higher compared to healthy lean control subjects [25]. The possible explanation for the association of L-MAFLD with metabolic risk factors is that BMI does not reflect the distribution of visceral adipose tissue, the source of excess free fatty acids (FFAs) which play a critical role in hepatic steatosis [22, 26]. So, our results augment the association of L-MAFLD with some components of metabolic syndrome, paying attention to this pathogenic mechanism, which could impact the management of individuals with L-MAFLD.

It is widely acknowledged that the most significant predictor of both overall and liver-related mortality in patients with MAFLD is the fibrosis stage [27]. In this study, 17.7% of MAFLD patients had significant fibrosis, they were predominantly males, with older age, having a higher BMI, metabolic risk factors, elevated liver enzymes, FIB-4, but lower platelets and HDL, all these factors associated with obese-MAFLD, of which male gender, diabetes, hypertension, higher level of ALT, ALP, Hs-CRP, Uric acid, and lower level of platelets and HDL-cholesterol were predictors of significant fibrosis in obese-MAFLD. Reports indicate that BMI and components of metabolic syndrome, along with demographic data, are key factors implicated in the development of hepatic steatosis and its progression to fibrosis [28], Diabetes is linked to a higher risk of advanced fibrosis, complications related to cirrhosis, and mortality from liver disease [29], several studies report that MAFLD with advanced fibrosis is found more frequently in diabetic patients [28, 30, 31], chronic hyperinsulinemia and hyperglycemia, and excessive rates of lipolysis from IR-adipose tissue in diabetes is driving force for the development of steatosis, and activate harmful inflammatory pathways with subsequent fibrosis [32, 33]. Age can predict advanced fibrosis in both lean and non-lean MASLD with age ≥ 65 [34]. Shao et al. showed that increased WC and BMI ≥ 28 kg/m^2^ plus ALT were predictors of fibrosis in overweight and obese patients, respectively [35].

There is a remarkable paucity of data on the differential risk factors of fibrosis between lean and obese MAFLD with different grades of fibrosis. One of the strengths of our study is that we compare the overall risk factors of fibrosis between individuals with significant and non-significant fibrosis in both lean and obese MAFLD using LSM, the results showed that L-MAFLD patients had a lower value of hepatic steatosis and degree of fibrosis compared to obese-MAFLD, however, using FIB-4, L-MAFLD had a higher degree of fibrosis, this higher value could be due to that L-MAFLD were more older than obese-MAFLD and having lower platelet count and ALT despite they were within the normal limits. There is contradictory data about the association of L-MAFLD patients with significant fibrosis, similar to our findings, it is reported that normal-weight individuals with NAFLD seem to have a low risk of advanced fibrosis [36], another study showed that L-NAFLD group had less advanced fibrosis, however, they had a higher overall mortality than patients with overweight/obese-NAFLD, despite having healthier metabolic profile [37], this indicate that L-MAFLD is not just a benign condition even in absence of significant fibrosis. On the other side, multiple studies showed that L-MAFLD had higher fibrosis scores and rates of cardiovascular morbidity and all-cause mortality, and fibrosis score in its advanced stages [38, 39], also using MRI elastography L-MAFLD had significant and advanced fibrosis similar to those in overweight/obese patients, but with associated metabolic risk factors [40]. This signifies that the presence of metabolic abnormality might have a significant effect on hepatic fibrosis even in lean individuals.

On grading the degree of fibrosis using LSM, the current study showed that L-MAFLD with significant fibrosis (≥ F2) had higher Hs-CRP level, AST and ALP compared to L-MAFLD with non-significant fibrosis, however, none of these factors could predict fibrosis, this could be due to the small sample size of L-MAFLD with significant fibrosis in this study.

Our study stands out in being multicentric, having a large sample size, and assessing factors associated with different grades of fibrosis in lean and obese-MAFLD patients. However, the study's limitations are that first, we didn’t assess the long-term follow-up of patients with L-MAFLD, cardiovascular, liver-related risks, and overall mortality. Second, our patients with significant fibrosis (≥ F2) should be further subclassified into significant and advanced fibrosis or cirrhosis. However, the statistical power might not be adequate for conducting logistic analysis due to the small sample size in the L-MAFLD subgroup. Finally, this study concentrated exclusively on the metabolic and imaging characteristics of the three groups, without examining gene polymorphisms or other metabolites that could predispose non-obese individuals to MAFLD.

In conclusion, despite having normal BMI, lean individuals may have metabolic risk factors that could progress to hepatic steatosis and fibrosis, however still obese-MAFLD have more metabolic risk factors and significant fibrosis than L-MAFLD, finally L-MAFLD is not just a benign state, that needs more studies to verify risk factors for fibrosis progression.

## Data Availability

No datasets were generated or analysed during the current study.
